# Clinical presentation, diagnostic investigations and follow-up of a Bengal tiger (*Panthera tigris tigris)* affected by ambulatory tetraparesis

**DOI:** 10.29374/2527-2179.bjvm008024

**Published:** 2025-01-08

**Authors:** Stefania Mosconi, Manuel Morici, Edoardo Auriemma, Salvatore Di Graci, Anna Calloni, Giordana Zanna, Federica Tirrito

**Affiliations:** 1 Veterinarian, Neurology Department, AniCura Istituto Veterinario di Novara, Granozzo con Monticello, Novara, Italy.; 2 Pombia Park S.r.l., Pombia, Italy.; 3 Veterinarian, Diagnostic Imaging Department, AniCura Istituto Veterinario di Novara, Granozzo con Monticello, Novara, Italy.; 4 Veterinarian, Anesthesiology Department, AniCura Istituto Veterinario di Novara, Granozzo con Monticello, Novara, Italy.; 5 Veterinarian, Dermatology Department, AniCura Istituto Veterinario di Novara, Granozzo con Monticello, Novara, Italy.; 6 Veterinarian, Studio Veterinario Associato Vet2Vet di Ferri e Porporato, Orbassano, Torino, Italy.

**Keywords:** brainstem auditory evoked potential test, computed tomography, felids, spondylomyelopathy, wildlife, exame do potencial evocado auditivo do tronco encefálico, tomografia computadorizada, felinos, espondilomielopatia, animais selvagens

## Abstract

An 11-year-old male Bengal tiger (*Panthera tigris tigris*) was referred for a 2-week history of ambulatory tetraparesis, generalized ataxia, and hypermetric gait, associated with mild right head tilt and spontaneous proprioceptive deficit on the right forelimb. Neuroanatomical localization was C1-C5 myelopathy; cerebellum-vestibular system involvement was also considered. Hematology and serum biochemistry were unremarkable, although serum vitamin A (0.11 mg/L) was below the reference range (0.17 - 0.36 mg/L). Indirect hemagglutination test for *Toxoplasma gondii* was positive (antibodies titer 1:640). Computed tomography of the head and cervical column showed hypertrophic degenerative remodeling of the vertebral articular joint processes, causing severe vertebral canal stenosis and bilateral spinal cord compression at C2-C3. In addition, bilateral otitis media was present, without signs of intracranial extension of the inflammation by imaging. Brainstem auditory evoked potential test revealed a partial, bilateral conductive deafness. Cerebrospinal fluid (CSF) analysis resulted normal; CSF PCR for *T. gondii* was negative. A diagnosis of osseous-associated cervical spondylomyelopathy (OA-CSM) and concurrent bilateral otitis media was obtained. Glucocorticoids, movement restriction, vitamin A supplementation, and clindamycin were instituted. Four weeks later the clinical signs deteriorated, and the animal was euthanized. To the authors’ knowledge this is the first report of OA-CSM in a tiger.

## Resumo

Um tigre-de-bengala macho de 11 anos (*Panthera tigris tigris*) foi referido por tetraparesia deambulatória, ataxia generalizada e marcha hipermétrica, associada a leve inclinação da cabeça para a direita e déficit proprioceptivo espontâneo no membro anterior direito há duas semanas. A localização neuroanatômica foi C1-C5; também foi considerado um envolvimento vestibulocerebelar. Análise hematobioquímica completa normal, a concentração sérica de vitamina A (0.11 mg/L) estava baixa (0.17 – 0.36 mg/L). Hemaglutinação indireta para *Toxoplasma gondii* foi positivo (1:640). A tomografia computadorizada pré e pós-contraste da cabeça e da coluna cervical mostrou remodelação degenerativa hipertrófica dos processos articulares, causando estenose severa do canal vertebral e compressão medular bilateral em C2-C3. Igualmente, presentava otite média bilateral e ausencia de sinais de imagem compativeis com extensão intracraniana da inflamação. O exame do potencial evocado auditivo do tronco encefálico revelou uma surdez parcial condutiva bilateral. Líquido cefalorraquidiano (LCR) normal; e PCR do LCR negativa para *T. gondii*. O diagnóstico foi espondilomielopatia cervical associada ao osso (OA-CSM) e otite média bilateral. Presceveram-se glicocorticoides, restrição de movimento, vitamina A e clindamicina. Após quatro semanas observou-se um declino clínicos e o animal foi eutanasiado. Pelo conhecimento dos autores, este é o primeiro relato de OA-CSM em um tigre.

## Introduction

Central nervous system pathologies in large captive non-domestic felids are currently poorly understood (Hecht et al., 2022). The scarce available information is based on post-mortem diagnosis, small case series with limited number of animals, or individual case reports (Hecht et al., 2022). The most frequent head and brain diseases are consistent with congenital/developmental anomalies; the most common is Chiari-like malformation associated with hypovitaminosis A, and inflammatory conditions such as otitis media and interna or meningoencephalitis due to intracranial extension of the inner ear inflammation, viral or mycotic etiologies, and parasite migration (Hecht et al., 2022). Other less frequent brain abnormalities include metabolic encephalopathies (i.e. degenerative leukoencephalopathy), traumatic events (i.e. fracture of the occipital condyle), neoplasia (i.e. meningioma), degenerative conditions (i.e. brain atrophy), and pituitary lesions (i.e. cystoadenoma, empty sella, nodular pituitary hyperplasia and pituitary hemorrhages) (Akin et al., 2013; Hecht et al., 2022; Moudgil et al., 2019; Ren et al., 2022).

Spinal injuries are related to congenital vertebral anomalies (i.e. vertebral dysplasia), vascular events (i.e. ischemic myelopathy), neoplastic disorders (i.e. multiple myeloma) and degenerative pathologies (i.e. subdural ossification) (Adaska & Lynch, 2004; Cushing et al., 2019; Hecht et al., 2022; Junginger et al., 2015).

However, the most commonly reported spinal problem of non-domestic felids is intervertebral disc disease (Hecht et al., 2022). A previous study on 37 large captive non-domestic felids identified degenerative disc conditions in 22% (8/37) of animals, including three lions (*P. leo*), four tigers (*P. tigris)*, and one leopard (*P. pardus*). The age of onset of clinical signs ranged between 8 and 19 years and neurological clinical signs included decreased activity, limb paresis, ataxia, and muscle atrophy. The diagnosis was often made by radiography and the majority of disc pathologies were located in the lumbar region, even if cervical and thoracic vertebrae were also involved (Kolmstetter et al., 2000; Wright et al., 2023). The disc diseases commonly reported in large captive felids include intervertebral disc extrusions, intervertebral disc protrusions, and vertebral spondylosis (Flegel et al., 2008; Kolmstetter et al., 2000; Lambrechts & Berry, 2000). The treatment of intervertebral disc herniation consists of medical management with corticosteroids and cage rest, or surgery (Flegel et al., 2008; Kolmstetter et al., 2000; Wright et al., 2023). Surgical procedures adopted in wild animals include thoracolumbar and lumbar dorsal hemilaminectomy, cervical hemilaminectomy, and ventral slot (Flegel et al., 2008; Ketz-Riley et al., 2004; Lambrechts & Berry, 2000; Wright et al., 2023). Factors associated with positive surgical outcomes in wild animals are represented by surgery performed before the deterioration of neurological conditions and post-operative movement restriction over several weeks (Flegel et al., 2008; Ketz-Riley et al., 2004; Lambrechts & Berry, 2000; Wright et al., 2023).

Recently, a single case report described the first case of disc-associated spondylomyelopathy in a 13-year-old Bengal tiger (*P. tigris tigris)* that was presented for gait abnormality with left ambulatory hemiparesis and spontaneous proprioceptive deficits in the left thoracic limb. In that case, a medical option was chosen with administration of corticosteroids and analgesics, along with movement restriction (Fugazzotto et al., 2022).

To the authors’ knowledge, no previous reports of osseous-associated cervical spondylomyelopathy (OA-CSM) have been reported in large captive non-domestic felids. The aim of this case report was to describe the clinical signs, diagnostic investigations, and outcome in a Bengal tiger (*P. tigris tigris)* affected by bilateral otitis media and OA-CSM.

## Case report

A 190 kg, 11-year-old captive neutered male Bengal tiger (*P. tigris tigris*) living at the Safari Park (Pombia, Italy) was presented to the neurology department of AniCura Istituto Veterinario di Novara (Granozzo con Monticello, Novara, Italy) for a 2-week history of progressive ambulatory abnormalities. The tiger was otherwise in good general clinical condition.

**Figure 1:**
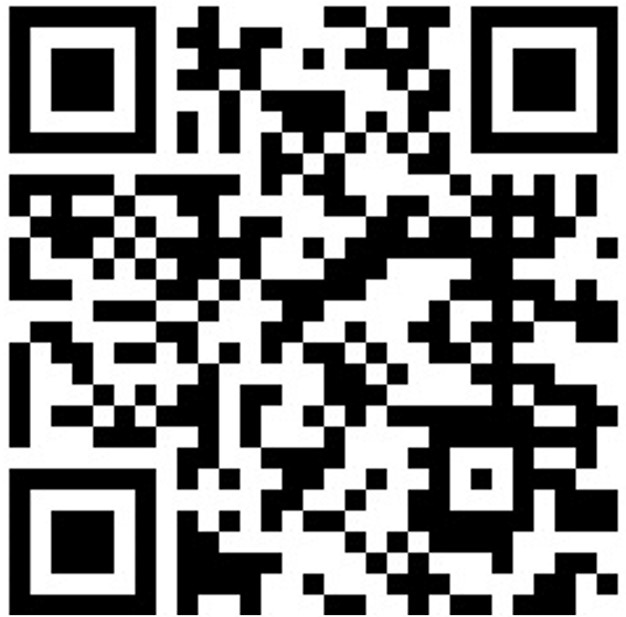
Scan the QR code to see the video of the tiger’s gait (Sigmapro, 2024).

The assessment of the tiger’s ambulation revealed generalized ataxia and hypermetric gait, associated with mild right head tilt and spontaneous proprioceptive deficit on the right forelimb (Figure 1). A complete neurological examination was not possible due to the aggressiveness of the animal.

Neuroanatomical localization was consistent with C1-C5 spinal cord segment myelopathy, but cerebellum-vestibular system involvement was also considered. The main differential diagnoses were spinal disc disease, degenerative disorder, or neoplastic pathology.

The Bengal tiger was sedated with dexmedetomidine (Dexdomitor, Vétoquinol Italia S.r.l., Bertinoro, Forlì-Cesena, Italy; 10 µg/kg, IM), ketamine (Nimatek, Dechra Veterinary Products S.r.l., Turin, Italy; 1.5 mg/kg, IM), and butorphanol (Dolorex, MSD Animal Health S.r.l., Segrate, Milan, Italy; 0.1 mg/kg, IM). General anesthesia was induced with propofol (Proposure, Boehringer Ingelheim Animal Health Italia S.p.a., Milan, Italy; EV) and then maintained with isoflurane (IsoFlo, Zoetis Italia S.r.l., Rome, Italy) in a mixture (50:50) of oxygen and medical air; the patient was mechanically ventilated (Mindray Wato ex-55, Shenzhen, China). The tiger was subjected to continuous electrocardiography, pulse oximetry, and non-invasive blood pressure monitoring.

During blood workup for general anesthesia, pre- and postcontrast computed tomography (CT) (64 slices, Optima 660, General Electric, Milan, Italy) study of the head and cervical column with the patient in dorsal recumbency was carried out; in addition video-otoscopy (Ub Cam Pro, Provix, Bucheon, Korea), brainstem auditory evoked response (BAER) test (Micromed, Mogliano Veneto, Treviso, Italy), and cerebrospinal fluid (CSF) analysis were performed.

Complete blood cell count, serum biochemistry (including renal, hepatic, pancreatic parameters and electrolytes analysis), and total T4 analysis were within normal limits, while serum vitamin A was slightly reduced (0.11 mg/L; range 0.17 - 0.36 mg/L) (Wheelhouse et al., 2015). Serology for *T. gondii* was indicative of parasitic exposure (indirect hemagglutination test, antibody titer 1:640; enzyme-linked immunosorbent assay, IgM: negative, IgG > 1:1024) (Greene, 2012).

**Figure 2:**
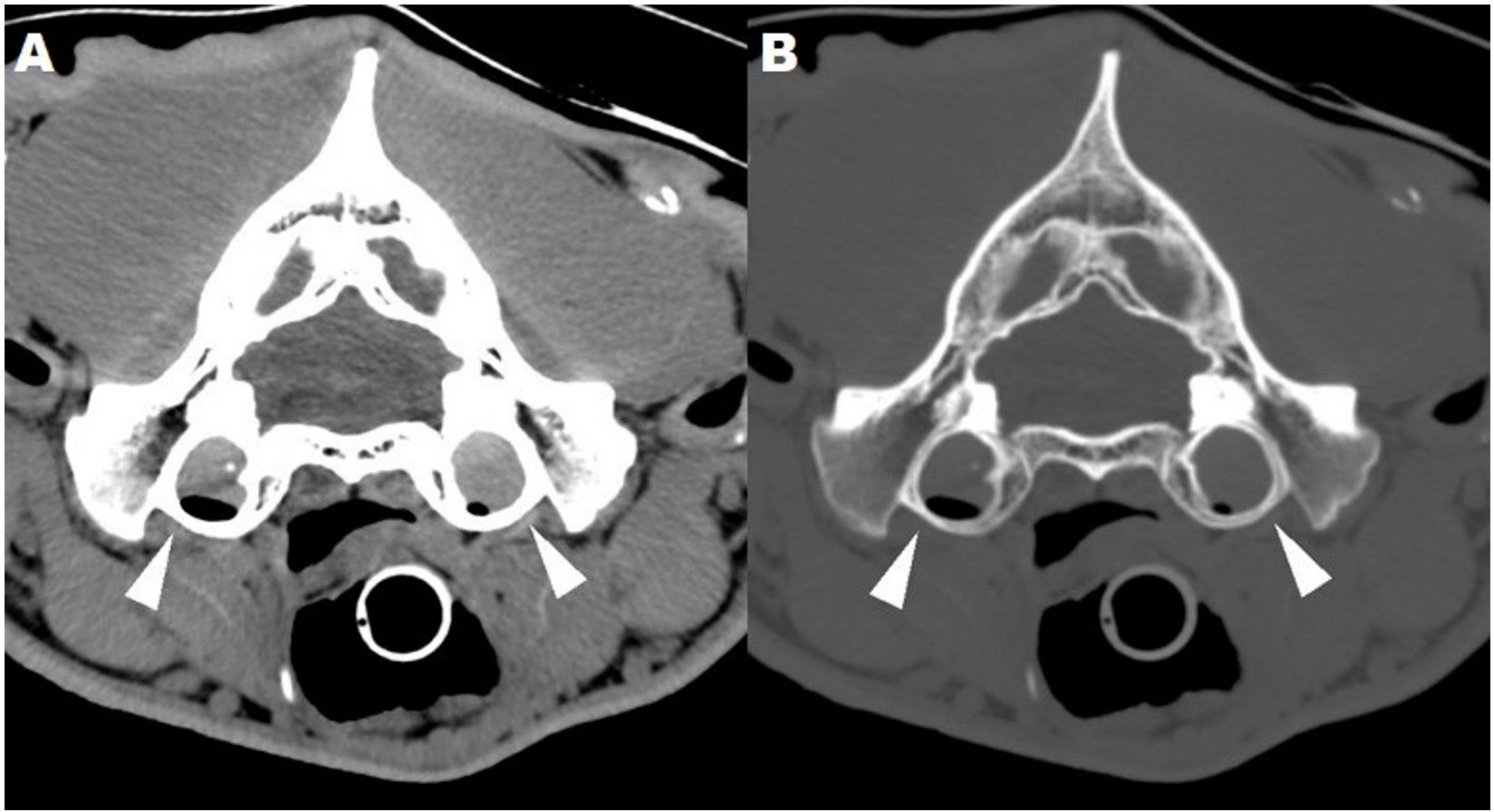
Transverse, post-contrast computed tomography images of the tiger’s head acquired in dorsal recumbency and displayed with soft tissue (A) and bone (B) windows. Note the bilateral otitis media (arrow heads) without evidence of intracranial extension of the pathological process.

**Figure 3:**
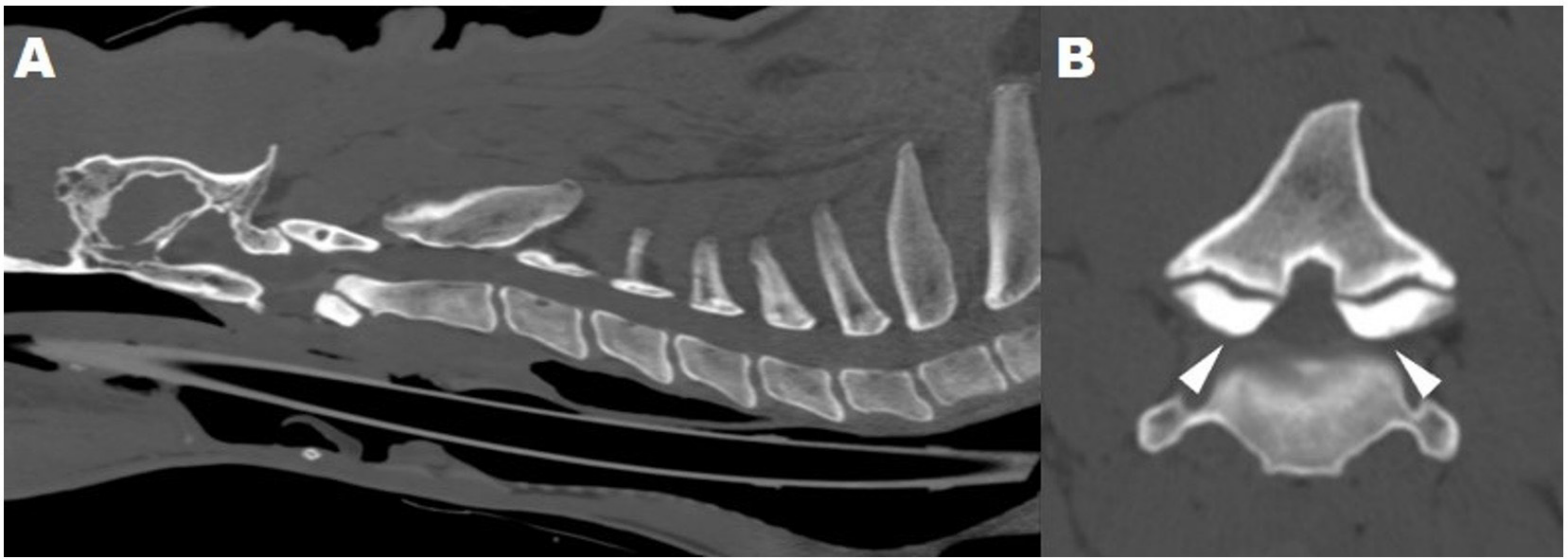
Sagittal (A) and transverse (B) post-contrast computed tomography images of the tiger’s cervical vertebral column, displayed with bone windows. Note the osseous-associated cervical spondylomyelopathy due to C2-C3 hypertrophic degenerative remodeling of the articular joint processes (arrow heads) causing vertebral canal stenosis and bilateral compression of the spinal cord that was focally misshaped.

CT of the head showed an accumulation of soft tissue non-enhancing material, obliterating the lumen of the tympanic bullae. These CT findings were consistent with bilateral otitis media, without evidence of intracranial extension of the suspected otogenic inflammatory-infectious disease (Figure 2). At the level of C2-C3 intervertebral disc space a bilateral hypertrophic remodeling of the articular joint processes was noted, causing vertebral canal stenosis and bilateral compression of the spinal cord, focally misshaped (Figure 3). These CT findings were compatible with a form of OA-CSM.

Video-otoscopy documented bilateral integrity of the tympanic membrane.

**Figure 4:**
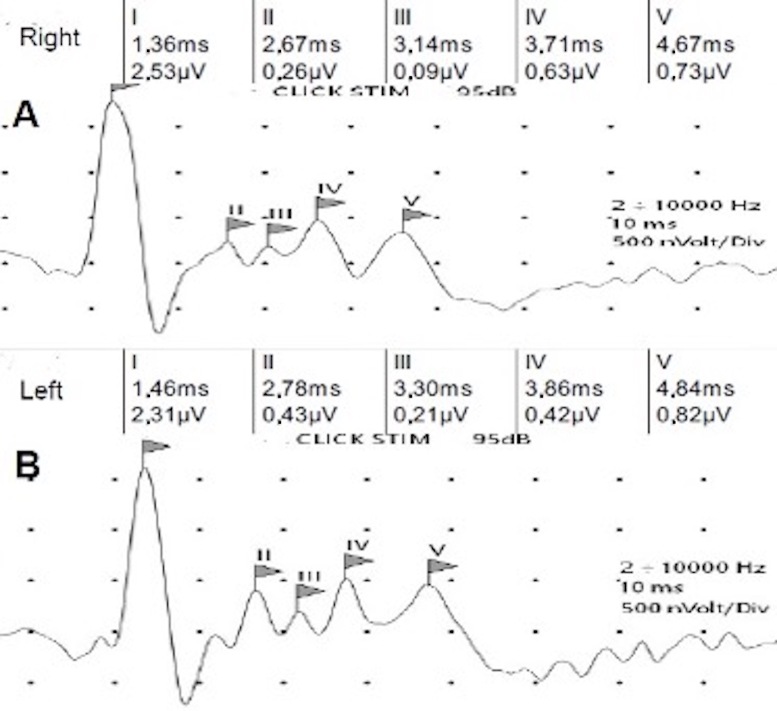
Brainstem auditory evoked response traces of the right (A) and left (B) ear, using a stimulus intensity of 95 dB. Note the generalized increase in latency and decrease in amplitude of all the waveforms (I to V).

Regarding the BAER test, stainless steel needles were inserted at the vertex (recording electrode), mastoid (reference electrode), and nuchal crest (ground electrode) (Cuddon, 2000). Acoustic stimuli (click) of increasing intensities (80, 90 and 95 dB) were produced and masking (white) noise at 30 dB less than the stimulus was delivered to the contralateral ear; artefact rejection was automatically performed (Cuddon, 2000). The waveforms corresponding to wave I to V were identified on the BAER traces of each ear, but a generalized increase in latency and decrease in amplitude of all the waveforms (I to V) was present in each trace examined and for each stimulus intensity; mild increase in the interpeak latency was also noticed in both ears (Figure 4). A partial, bilateral, acquired, conductive deafness due to bilateral otitis media was primarily suspected.

CSF was collected from the cerebellomedullary cistern and resulted normal with total nucleated cells count of 0 cells/µl and protein concentration of 18 mg/dL. CSF polymerase chain reaction (PCR) for *T. gondii* was performed and resulted negative.

Based on the findings of the described investigations, a diagnosis of bilateral otitis media associated with OA-CSM was made.

Treatment with prednisolone (Prednicortone, Dechra Veterinary Products S.r.l, Turin, Italy; 1 mg/kg orally every 24 hours for 10 days, then progressively tapered), movement restriction and vitamin A supplementation (Idrade, Fatro S.p.A., Ozzano dell’Emilia, Bologna, Italy; 2000 UI/kg once a week for four weeks and then every two weeks for four doses, IM) (Hartley et al., 2005) was started. Antibiotic treatment with clindamycin (Antirobe, Zoetis Italia S.r.l., Rome, Italy; 11 mg/kg orally every 24 hours) was added to treat the bilateral otitis and because of the serological positivity for *T. gondii*.

The initial follow-up after one week showed mild improvement of clinical conditions. However,-four weeks after diagnosis the neurological status deteriorated, the tiger became non-ambulatory tetraparetic, and was euthanized.

## Discussion

The CT findings of bilateral otitis media in this tiger were similar to those described for domestic small animals and documented soft tissue material within the tympanic bullae, without intracranial extension of the otogenic inflammatory-infectious disease (Belmudes et al., 2018; Garosi et al., 2001; Hecht et al., 2022).

The BAER test was consistent with partial, bilateral, acquired, conductive deafness that was probably due to otitis media. Similarities were found between our tracing and those previously reported in small animals with bilateral otitis media (Cuddon, 2000). To the authors’ knowledge, this is the first report of BAER test in an adult tiger affected by bilateral otitis media, and has only been described in cubs of Sumatran tigers (*P. tigris sumantrae*) affected by congenital vestibular syndrome that resulted normal (Wheelhouse et al., 2015). This diagnostic technique is minimally invasive, requires only sedation of the patient and consists of instruments that can be easily transported without moving the animals from their environment (Cuddon, 2000); for all of these aspects, the BAER test may be considered a valid option to assess the auditory pathway even in captive non-domestic felids.

CT is a rapid test for the visualization of the cervical spine and is useful in the evaluation of dogs affected by CSM (Costa, 2010). In our patient, imaging study allowed the diagnosis of OA-CSM with bilateral spinal cord compression at C2-C3 intervertebral space. The CT images were similar to those reported in dogs with OA-CSM and included irregular surface of the articular joint processes causing vertebral canal stenosis and bilateral spinal cord compression with loss of normal shape (Armstrong et al., 2014; Martin-Vaquero et al., 2014).

The etiology of cervical spondylomyelopathy in dogs is still unknown, but various hypotheses have been formulated including genetic, congenital, anatomical and nutritional causes (Costa, 2010). Dietary factors, such as overfeeding and excessive dietary calcium, may play an important role in the development of OA-CSM in giant dog breeds such as Great Danes (Costa, 2010). In captive non-domestic felids, vitamin A deficiency can result in skull and cervical vertebrae malformations, causing narrowing of the foramen magnum and thickening of the tentorium osseus cerebelli and dorsal arch of the atlas (Gross-Tsubery et al., 2020). In the present case, mild hypovitaminosis A was found, but a correlation between vitamin A deficiency and the OA-CSM was judged less probable due to the absence of calvarial hyperostosis and C1 vertebra abnormalities that were not identified on CT. Nevertheless, vitamin A was supplemented but without improvement of clinical symptoms.

The treatment of OA-CSM in dogs can either be conservative or surgical (Costa, 2010; Dewey & Costa, 2016). Medical therapy is usually the initial treatment and is considered effective to determine if the spinal cord lesions are reversible (Dewey & Costa, 2016). Medical management consists of corticosteroids at an anti-inflammatory dose and progressively tapered over the course of 2-3 weeks in association with exercise restriction (Dewey & Costa, 2016). In small animals, surgical treatment is considered on the basis of severity of neurologic signs, degree of pain, type and severity of compressive lesion, and response to medical management (Costa, 2010; Dewey & Costa, 2016). The rate of improvement after medical and surgical treatments for CSM in dogs is 54% and 81%, respectively (Costa et al., 2008). Likewise, survival time of dogs with CSM that were treated surgically (median and mean times: 36 and 46.5 months, respectively) was not significantly different from the survival of dogs treated medically (median and mean times: 36 and 48 months, respectively) (Costa et al., 2008). In our case, pharmacological treatment with corticosteroids and cage rest was prescribed, but neurological symptoms deteriorated and for this reason the tiger was euthanized. Because of the poor response of the animal to the conservative treatments and the possible difficulties in managing the tiger after diagnosis, decompressive surgery was not performed even if it would have been a treatment option.

## Conclusions

In conclusion, OA-CSM should be included in the differential diagnosis of tigers affected by myelopathies and CT may be needed to diagnose it.

Lastly, prognosis in captive non-domestic felids affected by OA-CSM and treated conservatively might be less favorable than what has been reported in small animals.
